# The Effects of Lutein and Zeaxanthin Supplementation on Brain Morphology in Older Adults: A Randomized, Controlled Trial

**DOI:** 10.1155/2019/3709402

**Published:** 2019-12-01

**Authors:** Catherine M. Mewborn, Cutter A. Lindbergh, B. Randy Hammond, Lisa M. Renzi-Hammond, L. Stephen Miller

**Affiliations:** ^1^Department of Psychology, University of Georgia, Athens, GA 30605, USA; ^2^Institute of Gerontology, Department of Health Promotions and Behavior, College of Public Health, University of Georgia, Athens, GA 30605, USA

## Abstract

A growing literature emphasizes the importance of lifestyle factors such as nutrition in successful aging. The current study examined if one year of supplementation with lutein (L) and zeaxanthin (Z), two nutrients with known antioxidative properties and cognitive benefits, impacted structural brain outcomes in older adults using a double-blind, randomized, placebo-controlled trial design. Community-dwelling older adults (20 males and 27 females) aged 65–87 years (*M* = 71.8 years, SD = 6.04 years) were randomized into supplement (*N* = 33) and placebo groups (*N* = 14) using simple randomization. The supplement group received 10 mg L + 2 mg Z daily for 12 months while the placebo group received a visually identical, inert placebo. L and Z were measured via retinal concentrations (macular pigment optical density or MPOD). Structural brain outcomes, focusing on global and frontal-temporal lobe regions, were acquired using both T1-weighted and DTI MRI sequences. We hypothesized that the supplement group would increase, maintain, or show attenuated loss in hypothesized regions-of-interest (ROIs) while the placebo group would show age-related declines in brain structural integrity over the course of the trial. While results showed age-related declines for frontal and temporal gray and white matter volumes, as well as fornix white matter microstructure across both groups, only minimal differences were found between the supplement and placebo groups. However, exploratory analyses showed that individuals who responded better to supplementation (i.e., showed greater increases in MPOD) showed less decline in global and prefrontal gray matter volume than supplement “nonresponders.” While results suggest that one year of L and Z supplementation may have limited effects on structural brain outcomes overall, there may be a subsample of individuals for whom supplementation of L and Z provides greater benefits. ClinicalTrials.gov number, NCT02023645.

## 1. Introduction

Aging is associated with many changes, both cognitive and neural, that contribute to negative outcomes such as decreased functional independence, significant personal and societal economic burden, and psychological distress for both aging individuals and their caregivers [1, 2]. One of the more common theories of biological aging is the Free Radical/Oxidative Stress Theory of Aging [3], which states that oxidative stress causes damage to DNA and proteins. In turn, oxidation leads to neural inflammation, neurotoxicity, reduced cerebral perfusion, and disruption of neural structure and cognitive functioning [4]. To combat the negative effects of oxidation, researchers have studied nutrients such as vitamins, flavonoids, and carotenoids for their potential in preventing and treating age-related cognitive and neural decline. Intake of these nutrients, along with healthy fatty acids and adherence to a balanced healthy diet, has been associated with positive neural effects, including preserved gray and white matter volume, white matter microstructure, and lower risk of cerebral infarcts, even after controlling for demographics and vascular risk factors [5–7].

Lutein (L) and zeaxanthin (Z) are two nutrients in the xanthophyll carotenoid family that have been suggested to benefit cognition and neural outcomes in older adults. Compared to other carotenoids, L and Z are the dominant carotenoids in the central nervous system (CNS) in both early- and late-life, where they account for 66–77% of the total carotenoid concentration in human brain tissue [8, 9]. Although the cognitive effects of L and Z have been well established and there is a growing literature on the direct neural effects, particularly regarding neural functioning and neural efficiency, much of what is known about the relation between L and Z and the brain has been determined through postmortem studies (e.g., [8–10]). Recent randomized control trials (RCTs) have demonstrated that the effects of L and Z supplementation can be measured at a neural level using functional neuroimaging technology [11]. However, there remains limited literature on the structural brain effects of L and Z, and, to our knowledge, the only published study that examined the effect of L and Z on brain structure in vivo was cross-sectional [12]. Thus, the aim of the current study was to extend previous literature on the relation between L and Z and brain structure in older adults by using a randomized, double-blind, placebo-controlled trial design to evaluate the impact of L and Z supplementation on several metrics of brain structure.

Specifically, we examined if one year of supplementation of L and Z impacted brain volume in older adults using T1-weighted sequences and white matter microstructure using diffusion tensor imaging (DTI) MRI sequences. We examined global measures of brain volume (i.e., global gray and white matter volume and white matter hypointensity volume) as well as specific regions-of-interest (ROIs) in the frontal and temporal lobes (i.e., prefrontal cortex, orbitofrontal cortex, anterior cingulate cortex, medial temporal cortex, and hippocampus), as gray and white matter volume declines are typically seen first in anterior regions of the brain in aging individuals (e.g., [13, 14]). We also examined global white matter microstructure and integrity of several anterior white matter tracts (i.e., genu of the corpus callosum, fornix, and anterior cingulum) that are particularly vulnerable to age-related decline (e.g., [15, 16]).

We hypothesized that L and Z supplementation would positively relate to brain structure such that the L and Z supplement group would increase, maintain, or show attenuated loss of their brain volume and white matter microstructure over the course of the trial while the placebo group would show age-related declines in brain volume and white matter microstructure (i.e., lower fractional anisotropy (FA), higher mean diffusivity (MD), and higher radial diffusivity (RD)); axial diffusivity (AD) was also examined as an exploratory measure of white matter microstructure but was not associated with directional hypotheses. Additionally, we hypothesized that L and Z supplementation would negatively relate to white matter hypointensity volume such that the supplement group was expected to maintain or attenuate increases of global white matter hypointensity volume, while the placebo group was expected to show age-related increases in these measures.

## 2. Materials and Methods

### 2.1. Participants

Community-dwelling older adults were recruited for participation in a year-long randomized, double-blind, placebo-controlled trial evaluating the impact of lutein (L) and zeaxanthin (Z) supplementation on vision, cognitive functioning, and neural integrity. Recruitment methods included newspaper advertisements, flyers, and electronic media (e.g., listservs). Exclusion criteria included macular degeneration, corrected visual acuity worse than 20 : 40, xanthophyll carotenoid supplementation within the six-month period prior to enrollment (with the exception of multivitamins that contained less than 1 mg L + Z/day), gastric conditions known to impair absorption of nutritional supplements (e.g., gastric bypass or gastric ulcer), left-handedness, traumatic brain injury, previous history of stroke, dementia, Parkinson's disease or other neurological condition known to impair cognitive function, and MRI incompatibility (e.g., cardiac pacemaker).

**Figure 1:**
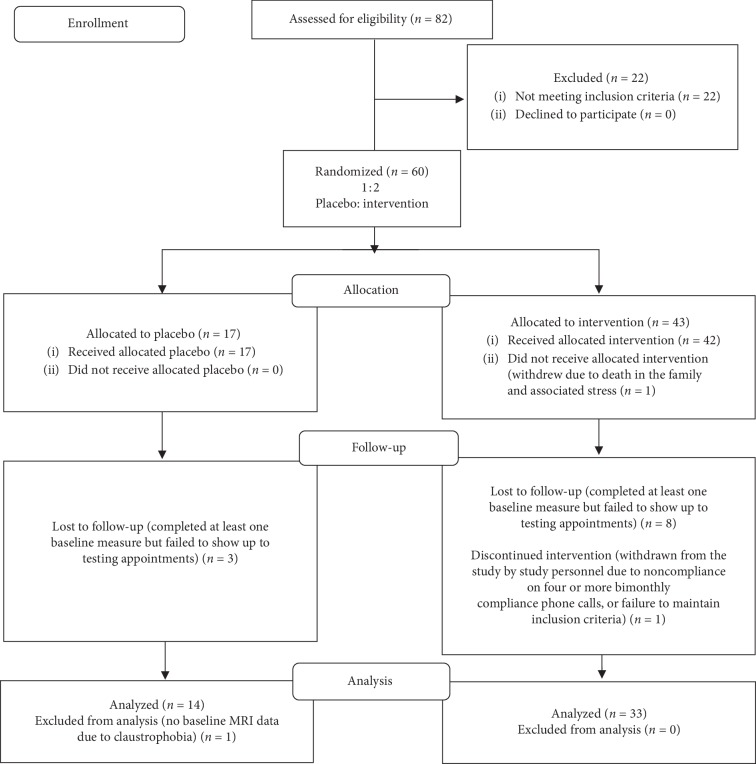
CONSORT flow diagram.

Sixty participants (23 males and 37 females), aged 65–92 years (*M* = 72.3 years, SD = 6.77 years), met the inclusion criteria and were randomized into either the active supplement group (*N* = 43) or the placebo group (*N* = 17). Of the 60 randomized participants, 47 participants (20 males and 27 females) aged 65–87 years (*M* = 71.8 years, SD = 6.04 years) completed the study, with the final sample size of 33 participants in the supplement group and 14 participants in the placebo group. A visual depiction of the study screening, randomization, intervention, and attrition process can be found in Figure 1, consistent with CONSORT guidelines [17].

### 2.2. Procedure

Eligible participants were randomly assigned to groups using a 2 : 1 active supplement to placebo group ratio. Simple randomization was conducted by the study coordinator, who was not involved in data collection. A master list of participant randomization was kept confidential by the study coordinator. All study personnel, including the staff who performed the assessments, were blinded to participant randomization throughout the course of the trial. Blinding was broken only after all data collection was complete and when necessary for statistical analysis of intervention effects.

Both the active supplement and placebo were provided by DSM Nutritional Products (Besel, Switzerland). The active supplement contained 10 mg L and 2 mg Z. The placebo was visually identical to the active supplement, and both the supplement and placebo were contained in identical, opaque, sealed bottles with labels that were visually identical except for the randomization code on the label. Thus, participants were also blinded to intervention condition. Participants were instructed to take one tablet from the bottle daily with a meal for 12 months.

Participants completed several preintervention visits to collect visual, cognitive, and neuroimaging measures. Participants also completed follow-up visits at 4 months and 8 months for ongoing data collection. Compliance to the intervention was monitored through twice monthly telephone calls and pill counts from bottles returned by the participants during follow-up visits. Participation could be discontinued by study personnel if individuals reported noncompliance on four or more of the telephone check-ins; however, no participants were withdrawn from the study due to noncompliance. Postintervention data were collected at 12 months and followed the same acquisition procedure as the preintervention data collection. Of note, although the larger RCT included a more extensive battery of outcome measures, the current project focused only on retinal L and Z data, together with structural neuroimaging data collected at pre- and postintervention visits. Results from other outcomes can be found in Lindbergh et al. and Hammond et al. [11, 18].

#### 2.2.1. Clinical Dementia Rating Scale (CDR)

Dementia severity was assessed using the Clinical Dementia Rating Scale [19] to confirm eligibility. The CDR is a semistructured interview conducted with both participants and collateral informants. The interviewer rates an individual's abilities in six cognitive and functional domains; scores from each of these domains are then combined to create a global rating of dementia severity ranging from 0 (no dementia) to 3 (severe dementia). A global score of 0.5 is often used as a proxy measure for mild cognitive impairment (MCI). Thus, to ensure the cognitive health of the sample, individuals who received a global rating of 0 or 0.5 were eligible for the study.

#### 2.2.2. Macular Pigment Optical Density (MPOD)

Retinal concentrations of L and Z were measured as macular pigment optical density (MPOD) and assessed using customized heterochromatic flicker photometry (cHFP). This method of data acquisition has been well validated as an in vivo measure of macular pigment density and has been fully described elsewhere (e.g., [20, 21]). Briefly, participants viewed a disc that was composed of two wavelengths of light (460 nanometer (nm) shortwave “blue” light and 570 nanometer (nm) midwave “green” light); the two wavelengths were presented in square-wave, counter-phase orientation, which caused the disc to appear to “flicker.” The task was customized to individual participants based on their critical flicker fusion frequency (CFF) values, which were measured in the same session. Participants then turned a knob to adjust the intensity of the 460 nm light until it appeared to match the luminance of the 570 nm light, causing the “flickering” to cease. This procedure was conducted in both the foveal and parafoveal regions of the retina. MPOD was calculated as the log of the intensity of 460 nm light required to match the 570 nm light in the fovea (where macular pigment is the densest) compared to the log of the intensity needed in the parafovea (where macular pigment is absent). MPOD data collection followed the same procedure at both pre- and postintervention visits.

### 2.3. Neuroimaging Acquisition

All images were acquired using a General Electric Signa HDx 3T MRI scanner (GE; Waukesha, WI, USA). A high-resolution 3D T1-weighted fast spoiled gradient echo (FSPGR) sequence was used to collect structural scans (TE = 3.2 ms; TR = 7.5 ms; flip angle = 20°; 154 axial slices; slice thickness = 1.2 mm; FOV = 256 × 256 mm in a 256 × 256 matrix). These images provided coverage from the top of the head to the brainstem, with a total acquisition time of 6 minutes and 20 seconds.

Diffusion weighted imaging (DWI) scans were acquired axially using a single-shot diffusion-weighted spin echo-EPI sequence. Slices covered from the top of the head to the brainstem and were acquired aligned to the anterior commissure-posterior commissure line. Scan parameters included: TE = 3.2 ms, TR = 15900 ms, 90° flip angle, 60 interleaved slices, slice gap = 0 mm, 2 mm isotropic voxels, acquisition matrix = 128 × 128, FOV = 256 × 256 mm, parallel acceleration factor = 2, *b*-value: 1000, and 30 optimized gradient directions with three b0 images. Total scan time for the DWI acquisition was 9 minutes and 38 seconds.

Additionally, two pairs of magnitude and phase images were acquired for fieldmap-based unwarping of DWIs (TE1 = 5.0 ms and TE2 = 7.2 ms, TR = 700 ms, 60 slices, slice gap = 0 mm, 2 mm isotropic voxels, acquisition matrix = 128 × 128, and FOV = 256 × 256 mm). Acquisition for each pair of images took approximately 2 minutes and 20 seconds. All neuroimaging acquisition followed the same procedure at both pre- and postintervention visits.

### 2.4. Neuroimaging Processing

#### 2.4.1. Brain Volume

**Figure 2:**
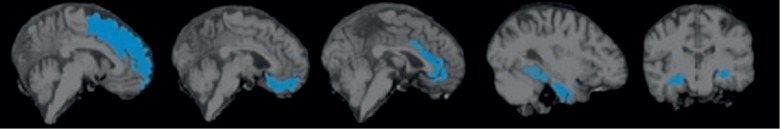
Volumetric regions-of-interest (ROIs). The figure depicts the masks used for volumetric ROI analyses from left to right: prefrontal cortex, orbitofrontal cortex, anterior cingulate cortex, medial temporal cortex, and hippocampus. Masks are superimposed on a representative T_1_-weighted image from a participant in our sample.

T1-weighted structural images were processed and segmented using FreeSurfer (v 6.0) (http://surfer.nmr.mgh.harvard.edu; [22]). Due to the longitudinal design of the study, the FreeSurfer longitudinal processing stream was utilized, which includes motion correction, skull stripping, automated transformation to Talairach space, normalization, and atlas registration, with processing of each time point initiated from a within-subjects template that represents mean subject anatomy across time points [23]. Following image processing, global gray matter, global white matter, and global white matter hypointensity volume (mm^3^) were extracted. The Desikan-Killiany atlas [24] was used to extract region-of-interest (ROI) volumes (see Figure 2), and all volumes were corrected for intracranial volume (ICV) prior to statistical analysis according to the formula: normalized volume = raw volume – *b* (ICV × mean ICV), where *b* is the slope of the regression of an ROI volume on ICV. When appropriate, right and left hemisphere values were summed to create a single value for each ROI.

#### 2.4.2. White Matter Microstructure

**Figure 3:**
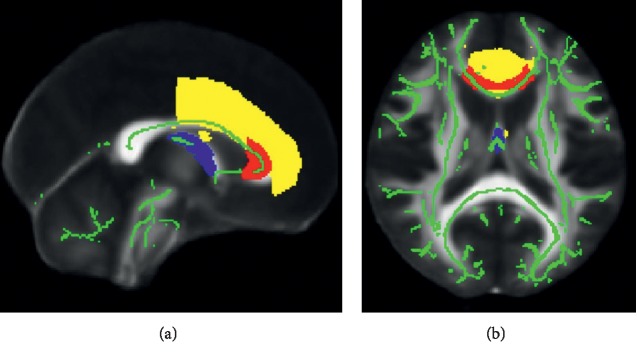
White matter microstructure regions-of-interest (ROIs). The figure depicts the masks used for white matter microstructure ROI analyses for the genu (red), fornix (blue), and anterior cingulum (yellow) in the sagittal view (a) and axial view (b). Masks are superimposed on a single-subject diffusion-weighted template in MNI space provided by Johns Hopkins University (JHU) in FMRIB's Software Library (FSL). The mean skeleton for the sample is overlaid on the diffusion-weighted image in bright green.

Diffusion weighted images (DWIs) were preprocessed using the Oxford Centre's Functional MRI of the Brain (FMRIB) Diffusion Toolbox (FTD) [25]. Preprocessing followed a standard pipeline, including head motion and eddy current correction, brain extraction, correction of distortion via fieldmap processing, and estimation of diffusion tensors for each voxel. Following preprocessing, Tract-Based Spatial Statistics (TBSS) [26] was used to optimize registration and create the mean diffusion images, which were thinned to create mean diffusion skeletons that represent the centers of all tracts common to the group of participants. The Johns Hopkins University (JHU) ICBM-DTI-81 White Matter Atlas [27] was used to create binary masks for each ROI, which were then multiplied with the diffusion skeletons to create skeletonized masks for each ROI (see Figure 3). We examined four standard diffusivity values for each ROI: fractional anisotropy (FA), mean diffusivity (MD), radial diffusivity (RD), and axial diffusivity (AD). Average diffusivity values were extracted from each skeletonized ROI and used in statistical analysis. When appropriate, right and left hemisphere values were summed to create a single mean value for each ROI.

### 2.5. Statistical Analysis

Baseline MPOD was used as a covariate in all analyses to ensure that intervention effects were not due to variations in baseline L and Z concentrations alone. Four individuals in our supplement group had possible MCI, as assessed by the CDR. We conducted analyses both with and without these participants and found no significant effect based on the removal of these outliers. Thus, in order to improve statistical power, we conducted final analyses with the whole sample, controlling for both age and baseline CDR scores, as age and level of cognitive impairment are strong predictors of brain structure in older adult populations (e.g., [13, 15]).

Changes in structural brain outcomes over time as a function of intervention condition were determined using analysis of covariance (ANCOVA). Global volumes (i.e., global gray matter, white matter, and white matter hypointensity volumes) and global diffusivity measures (i.e., global FA, RD, MD, and AD) were entered as dependent variables into a series of two-way mixed ANCOVAs with intervention group (active supplement vs. placebo) and timepoint (pre- vs. postintervention) as the independent variables and baseline age, CDR scores, and MPOD as the covariates.

Region-of-interest (ROI) outcomes were similarly entered together in groups of two-way mixed MANCOVAs with intervention group (active supplement vs. placebo) and timepoint (pre- vs. postintervention) as the independent variables and baseline age, CDR score, and MPOD as the covariates. The first group of analyses included ICV-corrected gray matter volume for orbitofrontal cortex, prefrontal cortex, anterior cingulate cortex, medial temporal cortex, and the hippocampus in the conglomerate dependent variable. The second group included ICV-corrected subcortical white matter volume of the orbitofrontal, prefrontal, anterior cingulate, and medial temporal cortices in the conglomerate dependent variable. The final group included white matter diffusivity values for the genu of the corpus callosum, fornix, and anterior cingulum in the conglomerate dependent variable. Analyses for each diffusivity parameter (i.e., FA, MD, RD, and AD) were performed separately. If the MANCOVAs reached significance, planned follow-up two-way mixed ANCOVA analyses were conducted to determine changes in specific ROIs as a function of intervention condition and timepoint, controlling for baseline age, CDR score, and MPOD values.

## 3. Results and Discussion

Demographic characteristics of the sample can be found in Table 1. Independent-samples *t*-tests confirmed that the supplement group and placebo group did not differ significantly at preintervention on age, education level, or baseline MPOD concentrations. Chi-square tests also confirmed that the two groups did not differ significantly at preintervention in terms of sex or level of cognitive impairment (CDR score). Similarly, there were no significant differences between participants who completed the study (*N* = 47) and participants who attrited (*N* = 13) with respect to age, sex, education, baseline MPOD, and cognitive impairment (CDR score).

### 3.1. Brain Volume

Pre- and postintervention values for both global and regional brain volumes can be found in Table 2. When controlling for baseline age, MPOD, and cognitive impairment (CDR score), there were no significant main effects of group for any of the measures of global brain volume. Additionally, there were no significant main effects of time, although both groups showed nonsignificant age-related changes in brain volumes (i.e., decreased global gray and white matter volume and white matter hypointensity volume). There were no significant group *∗* time interactions for any measures of global brain volume.

A two-way mixed MANCOVA showed a significant main effect of time for gray matter ROIs (i.e., gray matter volume of the prefrontal cortex, orbitofrontal cortex, anterior cingulate cortex, medial temporal lobe, and hippocampus) (*F* = 3.02, *p*=0.022), but no significant main effect of group or group *∗* time interaction was observed. When conducting planned follow-up ANCOVAs, only changes over time in medial temporal lobe volume, regardless of group status, were individually significant (*F* = 6.72, *p*=0.013). There were no significant changes over time, between group differences, or group *∗* time interactions for prefrontal cortex, orbitofrontal cortex, anterior cingulate cortex, or hippocampal volume.

Similarly, a two-way mixed MANCOVA showed a significant main effect of time for white matter ROIs (i.e., subcortical white matter volume of the prefrontal cortex, orbitofrontal cortex, anterior cingulate cortex, and medial temporal lobe) (*F* = 4.00, *p*=0.016), but no significant main effect of group or group *∗* time interaction. However, follow-up ANCOVAs showed that changes over time in the anterior cingulate cortex, regardless of group status, were individually significant (*F* = 6.19, *p*=0.017). No other significant main effects of time, main effects of group, or group *∗* time interactions were observed for subcortical prefrontal cortex, orbitofrontal cortex, or medial temporal lobe white matter volume.

### 3.2. White Matter Microstructure

Pre- and postintervention values for both global and regional white matter microstructure can be found in Table 3. When controlling for baseline age, MPOD, and cognitive impairment (CDR score), results showed a significant main effect of time for global FA (*F* = 5.31, *p*=0.004), but no significant main effect of group or group *∗* time interaction. Contrary to hypotheses, both groups showed a significant increase in global FA. There were no significant main effects of time for global RD, MD, or AD, and no significant group differences or group *∗* time interactions were observed.

Two-way mixed MANCOVAs for ROIs (i.e., genu of the corpus callosum, fornix, and anterior cingulum) showed a significant main effect of time for FA (*F* = 5.31, *p*=0.004), but no significant main effects of group or group *∗* time interactions. Follow-up ANCOVAs showed that changes over time in the anterior cingulum (*F* = 8.60, *p*=0.005) and fornix (*F* = 8.29, *p*=0.006), regardless of group status, were individually significant. No other significant main effects of time, main effects of group, or group *∗* time interactions were observed for genu FA and RD, MD, or AD values in the ROIs.

### 3.3. Intervention Response

To confirm intervention effectiveness, paired-samples *t*-tests were conducted in both the supplement group and placebo group to assess statistically significant changes in MPOD concentrations over the course of the intervention. Analyses confirmed that the supplement group showed a significant increase in MPOD (*t* = 2.27, *p*=0.030) over the course of the trial, while the placebo group did not show any significant changes in MPOD (*t* = 0.788, *p*=0.445). However, there was heterogeneity in both groups, with some individuals in the placebo group showing increased MPOD concentrations and some individuals in the supplement group appearing to fail to respond to intervention (i.e., showing stable or decreased MPOD concentrations). To explore this heterogeneity, individuals were classified into two categories: (1) those who showed an increase in MPOD of 0.10 + log units from pre- to postintervention were classified as “responders” and (2) all others who showed a stable, decreased, or nonsignificant increase (<0.10 log units) in MPOD from pre- to postintervention were classified as “nonresponders” (see Table 4). Then, exploratory analyses were undertaken to determine if there were any brain changes corresponding to increased L and Z concentrations within the supplement group (“intervention responders”). The same analyses were repeated in the placebo group to determine if increases in MPOD, in the absence of supplementation, were associated with brain structural changes. As with the primary analyses above, all exploratory analyses were controlled for baseline age, MPOD, and CDR scores.

Independent-samples *t*-tests confirmed that there was no significant difference between treatment “responders” and “nonresponders” in either group with respect to baseline age, education, and MPOD concentrations. Additionally, chi-square tests confirmed that responders and nonresponders were not different with regard to sex and cognitive impairment (CDR scores).

**Figure 4:**
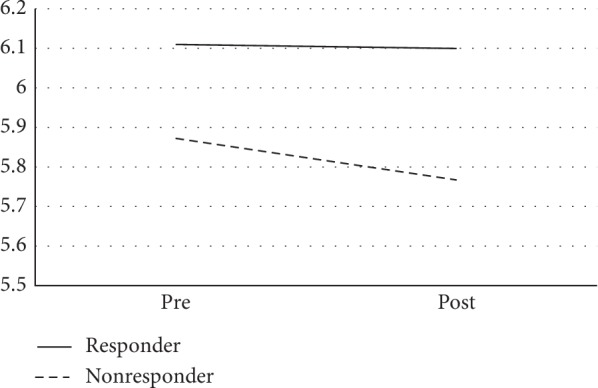
Changes in total gray matter volume. The figure shows changes in total gray matter volume (mm^3^ × 10^5^) between supplement group “responders” and “nonresponders.”

**Figure 5:**
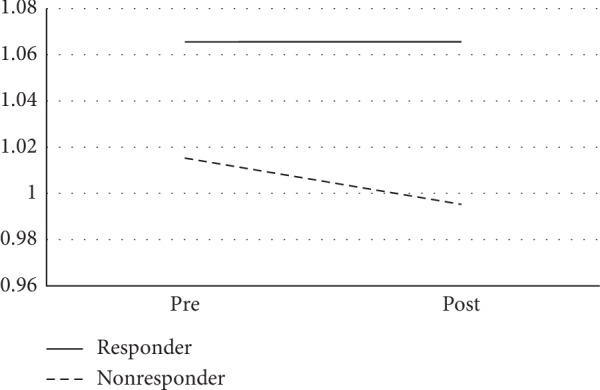
Changes in prefrontal cortex volume. The figure shows changes in total gray matter volume (mm^3^ × 10^5^) between supplement group “responders” and “nonresponders.”

In the supplement group, those who were classified as intervention responders (*N* = 15) showed significantly less decline in total gray matter volume (∆*R*^2^ = 0.144, *F* = 5.37, *p*=0.028) (see Figure 4) and prefrontal cortex gray matter volume (∆*R*^2^ = 0.125, *F* = 4.26, *p*=0.048) (see Figure 5) than those classified as nonresponders (*N* = 18). However, in the placebo group, there were no significant differences in any brain volume measures between individuals who showed increased MPOD (*N* = 7) versus those who showed stable or decreased MPOD (*N* = 7). There were also no significant differences in global or regional white matter microstructure measures between those classified as responders versus nonresponders in either the supplement or placebo groups.

## 4. Conclusions

The current study tested whether one year of supplementation with lutein (L) and zeaxanthin (Z), two xanthophyll carotenoids with known antioxidative properties and cognitive benefits, could prevent or slow age-related structural brain changes in a sample of community-dwelling older adults. Using a randomized, double-blind, placebo-controlled trial design, we hypothesized that older adults receiving supplementation with L and Z would increase, maintain, or show attenuated loss of brain volume and white matter microstructure in areas that are vulnerable to age-related decline (i.e., frontal and temporal gray and white matter regions) relative to older adults receiving a placebo.

Results showed expected age-related declines for frontal and medial-temporal gray and white matter, particularly medial temporal lobe gray matter and subcortical white matter of the anterior cingulate cortex, across both groups over the course of the trial. However, L and Z supplementation did not appear to influence this loss. No significant group differences or changes over time were observed in global brain volume outcomes (i.e., global gray matter, global white matter, and white matter hypointensity volume). Additionally, no interactions between group and time were found for any of the brain volume measures. Average percent change for both global volumes and frontal-temporal volumes ranged from less than 1% to 3%, consistent with previous estimates of the average annual rate of gray and white matter volume decline in older adults [14, 28].

We also did not find significant differences in white matter microstructure outcomes between the supplement and placebo groups. Results did show an average increase in global FA, across both groups. Significant changes were also seen in anterior white matter tracts, including increased FA in the anterior cingulum and an expected, age-related decline in fornix FA. While it is encouraging to see improvements in global and anterior cingulum white matter microstructure, it is important to note that these changes were seen in both the supplement and placebo groups and, thus, cannot be attributed to the intervention per se. Average percent change for both global and frontal-temporal white matter microstructure ranged from less than 1% to around 2%, again consistent with estimates of annual changes in diffusion metrics in healthy older adult populations [29, 30].

Although results confirmed that the intervention manipulation was effective (i.e., the supplement group showed a significant increase in retinal L and Z concentrations, while the placebo group did not), there was individual variability in both groups. This individual variability is not uncommon for nutritional intervention studies. There are many factors which may affect how one processes and absorbs nutritional supplements. For example, some studies have suggested that cholesterol levels and cosupplementation with other nutrients can impact the absorption and bioavailability of L and Z [31, 32]. Additionally, the very nature of being in a nutritional study may cause some participants to choose healthier and more nutritious foods, whether consciously or unconsciously. Thus, exploratory analyses were conducted to determine whether individuals who responded better to the intervention, or who otherwise increased their retinal L and Z concentration, showed better neural outcomes than those who showed no change or decreased retinal L and Z. While there were no differences in terms of white matter microstructure outcomes, results showed that in the supplement group, intervention “responders” (i.e., increased retinal L and Z) had significantly less decline in global and prefrontal gray matter volume than intervention “nonresponders” (i.e., stable or decreased retinal L and Z), even after controlling for factors such as baseline age, MPOD, and cognitive impairment. There were no concurrent differences observed in the placebo group. These results suggest that L and Z may slow age-related gray matter decline for a subset of individuals who appeared to reap greater benefit from the supplementation regimen.

As with any study, the present trial had limitations. For example, a similar prospective study on the effects of Vitamin B_12_ on brain volume found significant results but followed the older subjects for five years as opposed to only one [33]. Thus, longer time periods may be necessary to allow enough sensitivity to discriminate how dietary components influence morphological loss. Additionally, we did not have access to biomarkers, such as blood vitamin levels or lipid metabolites, in order to analyse the effect that these individual differences might have on intervention efficacy. Our sample size was small (e.g., the B_12_ study [33] tested 107 older adults) and suffered from considerable attrition (approximately 22%), which may have limited our power to detect certain group differences and intervention effects. Additionally, our sample was homogenous in terms of background, consisting of 100% Caucasian and predominantly cognitively healthy and highly educated individuals. Research has shown that more educated people also tend to have healthier diets [34], and our data support this trend. Baseline mean MPOD values for our sample were slightly higher than other published data for older adults [35, 36], suggesting that our sample may have consumed a more nutritious diet than the general population at baseline, prior to supplementation. In this respect, it is encouraging that a small dietary change provided benefit to even a subsample of individuals in our study who were already well nourished, educated, and affluent. Finally, as with any nutritional intervention, there was no true control condition; our entire sample has presumably been exposed to both L and Z throughout their lifetimes, and even those participants in the placebo condition continued to consume a normal diet for the duration of the trial, which likely contained some amount of L and Z which are naturally occurring in many foods. It is possible that the biggest effects of L and Z on the brain could be driven by deficiency.

While the current study found that one year of supplementation of L and Z had limited effects on the brain structural integrity of community-dwelling older adults, there is a growing body of literature to suggest that these nutrients are important for other aspects of cognitive and brain health. Other analyses from the current RCT have shown that supplementation with L and Z benefited cognition, particularly complex attention, cognitive flexibility [18], and verbal learning [11], as well as neural functioning in dorsolateral prefrontal cortex and anterior cingulate, areas that are vulnerable to age-related decline [11]. The importance of L and Z for cognitive and neural outcomes has been replicated cross-sectionally in other samples and by other researchers [9, 37–39] and is beginning to be confirmed longitudinally as well [40, 41].

The strength of this study lies in the novel approach used to investigate in vivo structural brain outcomes of a year-long, double-blind, placebo-controlled trial of lutein (L) and zeaxanthin (Z) supplementation in older adults. Exploratory analyses suggested that there was a small group of individuals who reaped greater benefit from L and Z supplementation and who showed less decline in global and prefrontal gray matter volume than individuals who did not appear to benefit from the intervention. To that end, future studies could explore factors which may impact the efficacy of L and Z supplementation, such as vascular health, cosupplementation with other nutrients, and other factors known to interact with nutrient bioavailability and absorption. Replication of this study in a larger and more diverse sample may also determine whether there are some individuals who could benefit even more from L and Z supplementation, such as individuals who are more cognitively impaired or have had less access to nutritious diets across their lifetime. Factors such as supplement dosage and particularly a longer intervention duration should also be considered when determining intervention efficacy. Finally, future studies will need to determine the long-term effects of L and Z supplementation on outcomes such as the development of dementia and accumulation of brain pathologies. While L and Z supplementation over one year appears to have limited effects on structural brain outcomes in older adults, it is likely not harmful [42] and appears to provide benefits for other aspects of cognitive and brain health that could improve or extend quality of life for older adults.

## Figures and Tables

**Table 1:** Preintervention characteristics.

	% or *M* (SD)		
	Supplement (*N* = 33)	Placebo (*N* = 14)	Overall sample (*N* = 47)
Age (years)	72.4 (6.27)	70.4 (5.43)	71.8 (6.04)
Sex (% female)	51.5%	71.4 %	57.4%
Race (% Caucasian)	100%	100%	100%
Education (years)	16.6 (3.31)	16.7 (3.02)	16.6 (3.19)
Cognitive impairment (%)			
No impairment (CDR = 0)	87.9%	100%	91.5%
Mild impairment (CDR = 0.5)	12.1%	0%	8.5%

Note: CDR = clinical dementia rating scale.

**Table 2:** Brain volume.

	*M* (SD) in mm^3^					
	Supplement (*N* = 33)		Placebo (*N* = 14)		Overall sample (*N* = 47)	
	Pre	Post	Pre	Post	Pre	Post
Gray matter						
Global	589258 (41075)	583090 (43892)	598888 (26886)	595608 (27435)	592127 (37387)	586819 (39829)
Regions-of-interest (ROIs)						^*∗*^
Prefrontal	102435 (10156)	101378 (10354)	105206 (7147)	104517 (6733)	103260 (9372)	102313 (9460)
Orbitofrontal	23839 (2388)	23809 (2208)	23430 (1645)	23590 (1719)	23717 (2184)	23744 (2058)
Anterior cingulate	7989 (1319)	7910 (1447)	7887 (1055)	7864 (1102)	7958 (1236)	7896 (1341)
Medial temporal	27361 (2589)	27234 (2558)	27063 (2342)	27234 (2658)	27212 (2496)	27234 (2559)
Hippocampus	8681 (989)	8407 (1050)	9144 (743)	8962 (886)	8819 (939)	8572 (1027)

White matter						
Global	437384 (74298)	432185 (81221)	427438 (40081)	423846 (39899)	434422 (65691)	429701 (71090)
Regions-of-interest (ROIs)						^*∗*^
Prefrontal	84364 (8774)	83736 (10028)	83119 (7414)	82317 (7317)	83994 (8332)	83313 (9247)
Orbitofrontal	18494 (2083)	18421 (2345)	18141 (1484)	18083 (1531)	18389 (1915)	18313 (9247)
Anterior cingulate	9228 (902)	9160 (951)	9336 (696)	9294 (699)	9260 (840)	9200 (878)^*∗*^
Medial temporal	17092 (2499)	16955 (2736)	16803 (2039)	16961 (2015)	17006 (2353)	16957 (2521)

Lateral ventricle	33250 (15696)	35066 (16500)	28855 (11638)	30229 (12088)	31941 (14622)	33625 (15352)
White matter hypointensities	7759 (14588)	8090 (16083)	5543 (4446)	5625 (4690)	7099 (12437)	7356 (13692)

Note: All volumes are corrected for intracranial volume (ICV) and have been rounded to the nearest mm^3^. ^*∗*^indicates a significant change from pre- to postintervention, *p* < 0.05, controlling for age and Clinical Dementia Rating (CDR) score.

**Table 3:** White matter microstructure.

	*M* (SD)					
	Supplement (*N* = 33)		Placebo (*N* = 14)		Overall sample (*N* = 47)	
	Pre	Post	Pre	Post	Pre	Post
Fractional anisotropy (FA)						
Global	0.55076 (0.02855)	0.55257 (0.03287)	0.55384 (0.01966)	0.56195 (0.02032)	0.55167 (0.02604)	0.55537 (0.02978)^*∗*^
Regions-of-interest (ROIs)						^*∗*^
Genu	0.61821 (.04982)	0.61228 (0.05182)	0.63634 (0.03231)	0.63892 (0.02889)	0.62361 (0.04573)	0.62021 (0.04750)
Anterior cingulum	0.62050 (0.05117)	0.62390 (0.05587)	0.63488 (0.03001)	0.64467 (0.03492)	0.62478 (0.04606)	0.63008 (0.05107)^*∗*^
Fornix	0.37296 (0.09130)	0.37186 (0.10075)	0.37936 (0.08800)	0.37860 (0.10308)	0.37487 (0.08942)	0.37387 (0.10037)^*∗*^
Radial diffusivity (RD)						
Global	0.00053(0.00005)	0.00053 (0.00061)	0.00051 (0.00003)	0.00050 (0.00003)	0.00053 (0.00005)	0.00052 (0.00006)
Regions-of-interest (ROIs)						
Genu	0.00050 (0.00010)	0.00052 (0.00010)	0.00047 (0.00049)	0.00047 (0.00042)	0.00049 (0.00009)	0.00050 (0.00009)
Anterior cingulum	0.00053 (0.00009)	0.00052 (0.00010)	0.00049 (0.00006)	0.00048 (0.00006)	0.00052 (0.00009)	0.00051 (0.00009)
Fornix	0.00156 (0.00040)	0.00158 (0.00043)	0.00154 (0.00047)	0.00156 (0.00045)	0.00155 (0.00041)	0.00157 (0.00043)
Mean diffusivity (MD)						
Global	0.00081 (0.00005)	0.00081 (0.00005)	0.00079 (0.00003)	0.00078 (0.00003)	0.00081 (0.00042)	0.00080 (0.00005)
Regions-of-interest (ROIs)						
Genu	0.00086 (0.00009)	0.00087 (0.00009)	0.00083 (0.00004)	0.00083 (0.00003)	0.00085 (0.00008)	0.00086 (0.00008)
Anterior cingulum	0.00090 (0.00008)	0.00090 (0.00008)	0.00087 (0.00005)	0.00085 (0.00004)	0.00089 (0.00007)	0.00088 (0.00007)
Fornix	0.00191 (0.00036)	0.00193 (0.00038)	0.00189 (0.00044)	0.00191 (0.00039)	0.00190 (0.00038)	0.00193 (0.00038)
Axial diffusivity (AD)						
Global	0.00138 (0.00004)	0.00138 (0.00005)	0.00135 (0.00004)	0.00134 (0.00003)	0.00137 (0.00004)	0.00137 (0.00005)
Regions-of-interest (ROIs)						
Genu	0.00156 (0.00010)	0.00158 (0.00008)	0.00155 (0.00004)	0.00155 (0.00003)	0.00156 (0.00008)	0.00157 (0.00007)
Anterior cingulum	0.00164 (0.00007)	0.00166 (0.00006)	0.00161 (0.00005)	0.00160 (0.00003)	0.00163 (0.00006)	0.00164 (0.00006)
Fornix	0.00261 (0.00029)	0.00264 (0.00301)	0.00260 (0.00038)	0.00261 (0.00030)	0.00260 (0.00032)	0.00263 (0.00030)

Note: ^*∗*^indicates a significant change from pre- to postintervention, *p*=0.05, controlling for age and Clinical Dementia Rating (CDR) score.

**Table 4:** Treatment response–change in MPOD concentrations.

	*M* (SD)	
	Preintervention	Postintervention
Supplement group		
Responder (*N* = 15)	0.4547 (0.1992)	0.6947 (0.2329)^*∗*^
Nonresponder (*N* = 18)	0.5717 (0.1658)	0.5111 (0.1705)^*∗*^
Placebo group	0.4414 (0.0373)	0.4843 (0.0536)^*∗*^
Responder (*N* = 7)	0.3871 (0.1602)	0.5943 (0.1605)^*∗*^
Nonresponder (*N* = 7)	0.4957 (0.9888)	0.3743 (0.1820)
Total sample	0.4955 (0.0259)	0.5617 (0.0317)^*∗*^
Responder (*N* = 22)	0.4332 (0.1866)	0.6627 (0.2141)^*∗*^
Nonresponder (*N* = 25)	0.5504 (0.1521)	0.4728 (0.1811)^*∗*^

Note: ^*∗*^indicates a significant change from pre- to postintervention of *p* < 0.05.

## Data Availability

The data used to support the findings of this study are available from the corresponding author upon request.
